# The diverse dependence of galectin-1 and -8 on multivalency for the modulation of FGFR1 endocytosis

**DOI:** 10.1186/s12964-024-01661-3

**Published:** 2024-05-15

**Authors:** Dominika Żukowska, Aleksandra Chorążewska, Krzysztof Ciura, Aleksandra Gędaj, Marta Kalka, Marta Poźniak, Natalia Porębska, Łukasz Opaliński

**Affiliations:** Department of Protein Engineering, Faculty of Biotechnology, University of Wroclaw, Joliot-Curie 14a, Wroclaw, 50-383 Poland

**Keywords:** FGFR1, Galectins, Multivalency, Signaling, Endocytosis, CME

## Abstract

Fibroblast growth factor receptor 1 (FGFR1) is a N-glycosylated cell surface receptor tyrosine kinase, which upon recognition of specific extracellular ligands, fibroblast growth factors (FGFs), initiates an intracellular signaling. FGFR1 signaling ensures homeostasis of cells by fine-tuning essential cellular processes, like differentiation, division, motility and death. FGFR1 activity is coordinated at multiple steps and unbalanced FGFR1 signaling contributes to developmental diseases and cancers. One of the crucial control mechanisms over FGFR1 signaling is receptor endocytosis, which allows for rapid targeting of FGF-activated FGFR1 to lysosomes for degradation and the signal termination. We have recently demonstrated that N-glycans of FGFR1 are recognized by a precise set of extracellular galectins, secreted and intracellular multivalent lectins implicated in a plethora of cellular processes and altered in immune responses and cancers. Specific galectins trigger FGFR1 clustering, resulting in activation of the receptor and in initiation of intracellular signaling cascades that shape the cell physiology. Although some of galectin family members emerged recently as key players in the clathrin-independent endocytosis of specific cargoes, their impact on endocytosis of FGFR1 was largely unknown.

Here we assessed the contribution of extracellular galectins to the cellular uptake of FGFR1. We demonstrate that only galectin-1 induces internalization of FGFR1, whereas the majority of galectins predominantly inhibit endocytosis of the receptor. We focused on three representative galectins: galectin-1, -7 and -8 and we demonstrate that although all these galectins directly activate FGFR1 by the receptor crosslinking mechanism, they exert different effects on FGFR1 endocytosis. Galectin-1-mediated internalization of FGFR1 doesn’t require galectin-1 multivalency and occurs via clathrin-mediated endocytosis, resembling in this way the uptake of FGF/FGFR1 complex. In contrast galectin-7 and -8 impede FGFR1 endocytosis, causing stabilization of the receptor on the cell surface and prolonged propagation of the signals. Furthermore, using protein engineering approaches we demonstrate that it is possible to modulate or even fully reverse the endocytic potential of galectins.

**Supplementary Information:**

The online version contains supplementary material available at 10.1186/s12964-024-01661-3.

## Introduction

Fibroblast growth factor receptor 1 (FGFR1) is a class V receptor tyrosine kinase (RTK), which together with its canonical ligands, fibroblast growth factors (FGFs), form complex signaling hubs at the cell surface. FGF/FGFR enable transmission of signals from the extracellular environment to the cell interior and by this shape the cell and organism performance. In its extracellularly-oriented N-terminal region, FGFR1 includes three immunoglobulin-like domains (D1-D3) from which the D1 plays regulatory role, whereas the D2 and the D3 are involved in the binding of FGFs [1–7]. The extracellular part of FGFR1 contains also a stretch of negatively charged residues, so called the acidic box (AB) that prevents the receptor autoactivation in the absence of ligands [8]. FGFR1 is embedded in the plasma membrane via a single hydrophobic α-helix. From the cytosolic side, FGFR1 includes a regulatory juxtamembrane domain (JM) and a split tyrosine kinase (TK) directly involved in the initiation of intracellular phosphorylation events. Binding of FGFs to D2-D3 domains initiates FGFR1 dimerization and conformational changes within the intracellular portion of the receptor, resulting in the activation of TK and subsequent FGFR1 autophosphorylation [9, 10]. FGFR1 signaling, by controlling fundamental cellular processes like apoptosis, differentiation, division and motility, is critical for the human life at all its stages and its dysregulation is observed in numerous tumors including lung, breast, head and neck and urothelial cancers [10–17]. Several regulatory mechanisms are engaged in balancing FGFR1 signals such as endocytosis, phosphatases, negative regulatory proteins and feedback signaling [18].

Endocytosis is a complex process in which extracellular and cell surface cargoes, like proteins or lipids are sorted into vesicles and internalized into cells [19]. The endocytosis of macromolecules occurs via several distinct endocytic pathways, which ultimately lead to a cargo degradation in lysosomes or a cargo recycling to the plasma membrane [20–23]. Endocytic processes are often dysregulated in cancers and may significantly contribute to the retention of RTKs on the cell surface, enhancing the amplitude and duration of transmitted signals and thus facilitating oncogenic processes [19, 24]. Several reports indicated that endocytosis of FGFR1 in the complex with FGFs occurs mainly via clathrin medited endocytosis (CME), however clathrin-independent endocytosis (CIE) of FGFR1 was also detected and occurs through the caveolin-mediated pathway or macropinocytosis [18, 25–30]. FGF ligand type as well as specific accessory proteins regulate FGFR1 endocytosis [30–37]. Still, the precise signals for the initiation of FGFR1 internalization and for the selection of particular endocytic pathway are still elusive. It seems that the presence of the AP2 binding motifs in the intracellular tail of FGFR1, receptor dimerization, activation and triggered intracellular signaling may play crucial roles in FGFR1 CME [18, 26, 38, 39]. We have recently demonstrated that the spatial organization of FGFR1 on the cell surface may constitute the trigger for receptor internalization and affects the selection of engaged endocytic pathway. While FGFR1 dimerization mainly induces CME, FGFR1 clustering into larger complexes of distinct architectures simultaneously induces CME and CIE routes that depend on dynamin-2, resulting in rapid and highly efficient endocytosis of the receptor [26, 40–43].

In the extracellular part FGFR1 contains eight N-glycosylation motifs that are spread throughout the D1 (two sites), the D2 (two sites) and the D3 (four sites) domains, whose function, besides modulation of FGF and heparin binding, was for long largely unknown [44, 45]. We have recently demonstrated that N-glycans of FGFR1 constitute an additional layer of information, which is read by the extracellular lectins—galectins and used to alter spatial distribution of the receptor [46, 47]. Galectins comprise a family of 11 proteins in human that share the presence of the carbohydrate recognition domain (CRD), enabling binding of β-galactoside containing sugar chains. Human galectins are grouped into three subfamilies depending on their molecular architecture: prototype galectins (galectin-1, -2, -7, -10, -13 and -14) contain single CRD that may form dimers, a chimeric galectin-3 containing a single CRD and an N-terminal extension that facilitates oligomerization, and liquid–liquid phase separation, and tandem-repeat galectins (galectin-4, -8, -9 and -12) containing two different CRDs on a single polypeptide chain [48, 49]. Some members of galectin family are widely expressed in human body, whereas other galectins are present predominantly in specific tissues [50]. Galectins are capable of multivalent binding to N-glycan-bearing glycoproteins and glycolipids, and significantly contribute to development of diverse cancers [51–54]. Growing body of evidence positions galectins as major endocytic regulators of diverse cell surface proteins, including RTKs [45, 55]. Galectins can either inhibit uptake of cell surface proteins by trapping them in a galectin lattice, or promote endocytosis of specific glycosylated cargoes by the clathrin-independent GL-Lect mechanism [55–57].

We have recently shown that a precise set of galectins (galectin-1, -3, -7 and -8) binds N-glycans of the D3 domain of FGFR1, causing the differential receptor clustering, activation of the receptor, and initiation of the downstream signaling cascades that ultimately fine-tune the cell fate [47]. Importantly, we have demonstrated that multivalency of galectins is critical for the galectin-induced FGFR1 clustering and activation [47].

Our recent data indicated a possible important role of selected galectin family members in the endocytosis of FGFR1 [46]. However, the comprehensive analyses of the impact of the extracellular galectins on FGFR1 endocytosis has not been conducted to date. Also, the significance of galectins multivalency for their endocytic activity was unknown. These critical roles on galectins were assessed in this study.

## Results

### Extracellular galectins are internalized together with FGFR1

We have recently performed screening tests for the interaction between members of the human galectin family and FGFRs [47]. Using galectin dot blot arrays, pull-down and biolayer interferometry (BLI) we have shown that galectin-1, -3, -7 and -8 directly bind N-glycans of the D3 domain of FGFR1, induce FGFR1 clustering and subsequent receptor activation [47]. Therefore, in this study we aimed on elucidation whether these galectins, by direct binding to FGFR1 and receptor crosslinking, can affect endocytosis of the receptor. Since galectin family members not identified by us as direct FGFR1 interactors (galectin-2, -4, -9, -10, -13, -14 and -16) can still contribute to FGFR1 internalization in an indirect manner (e.g., by influencing the cell membrane characteristics or by binding FGFR1 partners), we decided to study these proteins as well.

**Fig. 1:**
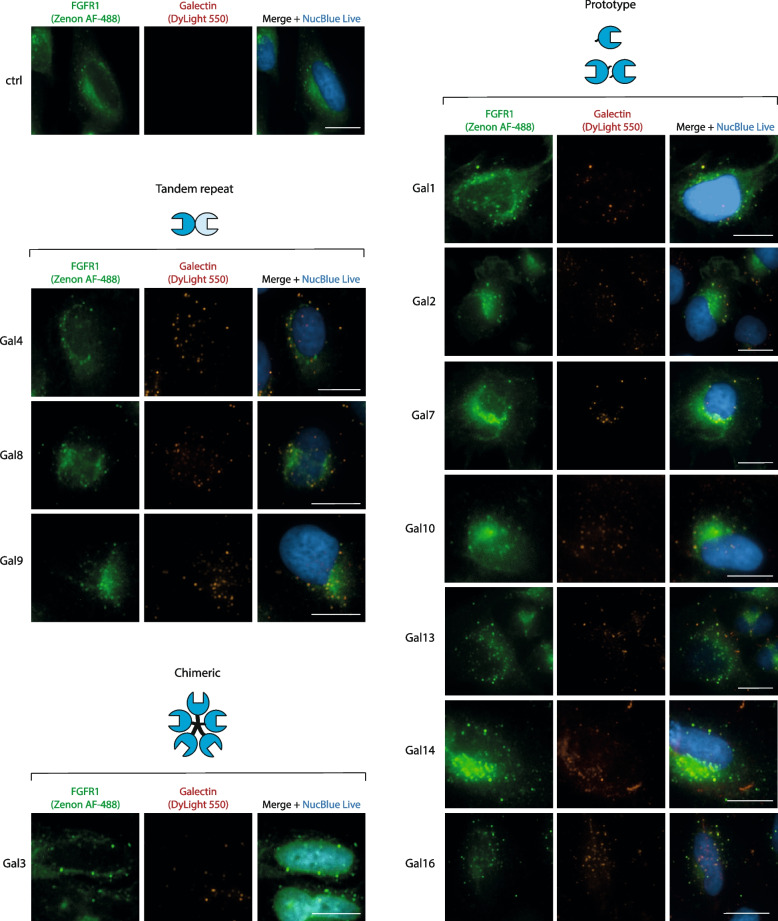
Co-localization of galectins with FGFR1. For analysis of galectins co-localization with FGFR1, fluorescently labeled galectins (20 μg/mL) were added to the lactose-treated U2OS-R1 cells for 30 min at 37 °C. Nuclei were stained with NucBlue Live. Cells were fixed and FGFR1 was visualized with T-Fc antibody and Zenon AF-488 using fluorescence microscopy. Representative images from three independent replicates are shown. Scale bars represent 20 μm

**Fig. 2:**
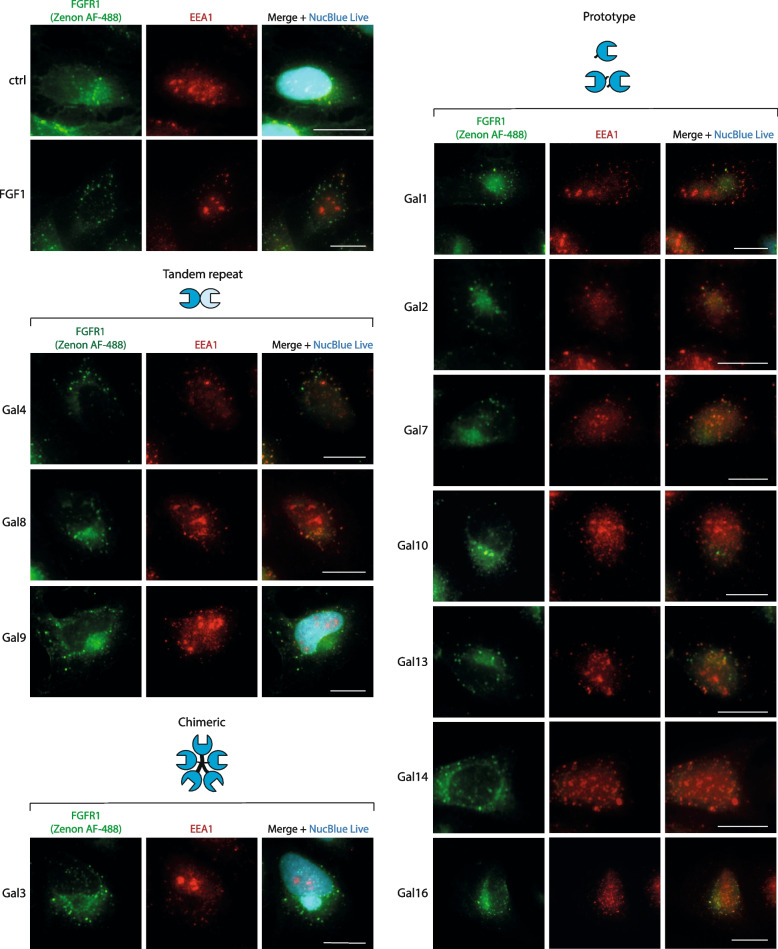
Co-localization of FGFR1 with an early endosome marker EEA1. U2OS-R1 cells were incubated with galectins (20 μg/mL) or FGF1 (100 ng/mL) for 30 min at 37 °C. Early endosomes were detected with rabbit anti-human polyclonal antibody specific for early endosome antigen 1 (EEA1) and anti-rabbit IgG secondary antibody conjugated to Alexa Fluor 594 (red). Nuclei were stained with NucBlue Live (blue). FGFR1 was visualized with T-Fc and Zenon AF-488 (green) using fluorescence microscopy. Experiments were performed in three independent replicates; representative images are shown. Scale bars represent 20 μm

We produced recombinant human galectins and fluorescently labeled these proteins with DyLight_550_. Galectins were then incubated for 30 min with lactose-treated U2OS-R1 cells and co-localization of galectins with FGFR1 was assessed with fluorescence microscopy. To be able to detect the effect of a particular galectin on FGFR1 endocytosis, we decided to deplete the cell surface of U2OS-R1 cells of undefined endogenous galectin mixture by washing cells with lactose prior incubation with studied galectin. Interestingly, for all tested galectins we observed an intracellular punctate signal of DyLight_550_ largely co-localizing with the signal specific for FGFR1 (Fig. 1). To study if the galectin-positive intracellular spots represent FGFR1-loaded endosomes, we assessed the co-localization of FGFR1 with an early endosome marker, EEA1. As shown in Fig. 2, FGFR1 co-localized with EEA1 after 30 min of treatment with non-labelled galectins. These data indicate that all studied galectins, no matter whether they directly bind FGFR1 or not, are partially internalized together with FGFR1.

### Extracellular galectins differentially modulate FGFR1 endocytosis

**Fig. 3:**
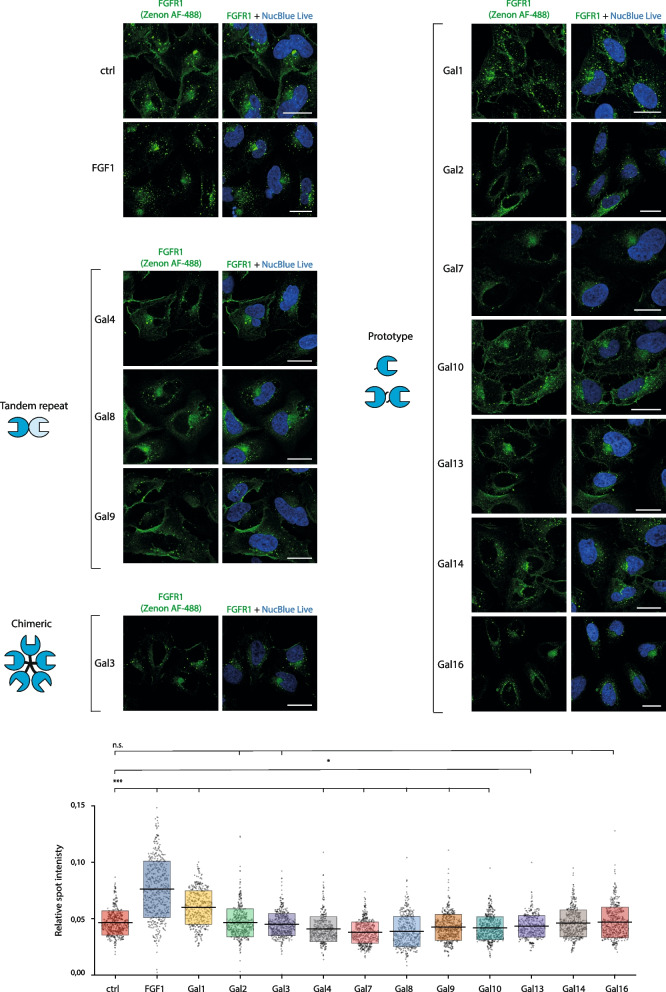
The differential impact of recombinant galectins on FGFR1 internalization. Serum starved lactose-treated U2OS-R1 cells were incubated with recombinant galectins (20 μg/mL) or FGF1 (100 ng/mL) for 30 min at 37 °C. Nuclei were stained with NucBlue Live dye, Zenon AF-488 was used for detection of FGFR1. Cells were analyzed with the quantitative confocal microscopy using Opera Phenix Plus platform. Representative images from three independent experiments are shown. Scale bars represent 20 μm. Each grey spot in the graph represents relative intracellular punctate signal intensity of FGFR1 in the single cell. At least 419 cells for each condition from three independent experiments were measured. Horizontal lines in the graph represent average intensity of intracellular FGFR1 punctate signal, whereas boxes represent ± SD. Statistical analyses were performed with Kruskal–Wallis H test (*p < 0.05; **p < 0.005 and ***p < 0.001)

To study if galectins exert any effect on the efficiency of FGFR1 internalization, or they are just passively endocytosed during the basal uptake of FGFR1, we employed the high content quantitative confocal microscopy. Lactose-washed U2OS-R1 cells were treated with FGF1 (positive control, inducing CME of FGFR1) or recombinant galectins and the intensity of the intracellular FGFR1 punctate signal was measured [26, 38]. As shown in Fig. 3, the supplementation of cells with FGF1 resulted in largely enhanced intracellular signal of FGFR1, as expected. Galectin-1 treatment resulted in the significantly increased intracellular signal of FGFR1, however the observed effect was about 50% lower than the one seen for FGF1 (Fig. 3). For galectin-2, -3, -14 and -16 was haven’t observed any significant impact on the cellular uptake of FGFR1 (Fig. 3). Interestingly, the supplementation of cells with galectin-4, -7, -8, -9, -10, -13 resulted in a significantly reduced levels of the intracellular punctate signal of FGFR1, with the strongest effect measured for galectin-7 and -8 (Fig. 3).

**Fig. 4:**
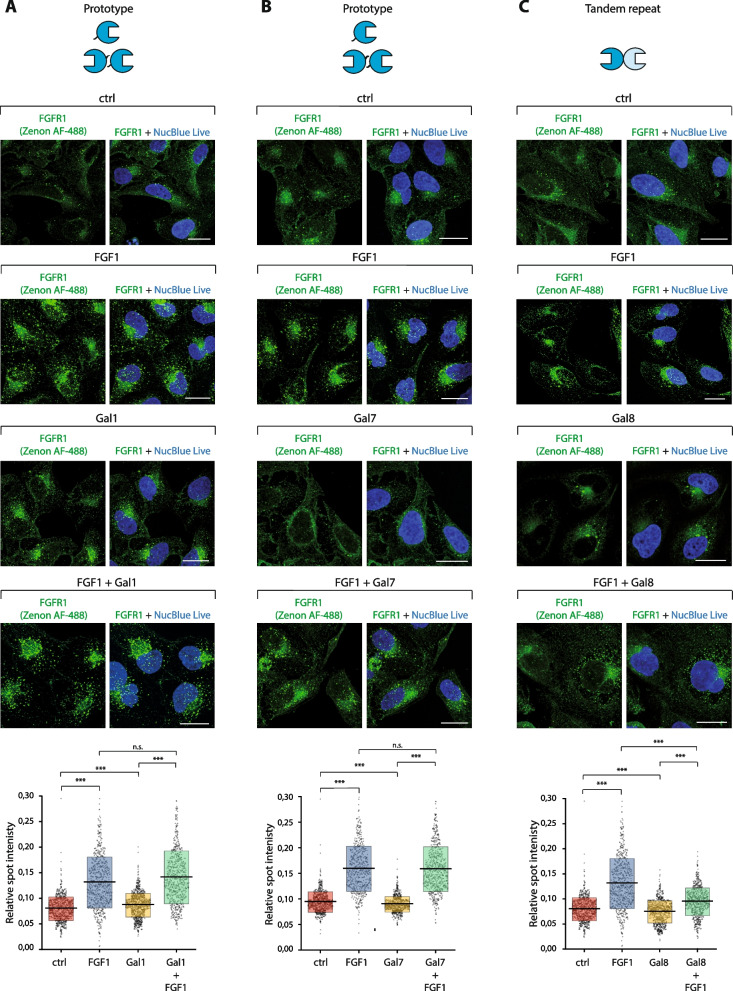
Effects of galectins on FGF1-induced FGFR1 internalization. Lactose-washed U2OS-R1 cells were incubated with recombinant galectins (20 μg/mL), FGF1 (100 ng/mL) or mixtures of studied proteins for 30 min at 37 °C. Nuclei were stained with NucBlue Live dye, T-Fc and Zenon AF-488 were used for detection of FGFR1. Cells were analyzed with the quantitative confocal microscopy. Representative images from at least three independent experiments are shown. Scale bars represent 20 μm. Each grey spot in the graph represents relative intracellular punctate signal intensity of FGFR1 in the single cell. At least 573 cells for each condition from three independent experiments were measured. Horizontal lines in the graph represent average intensity of intracellular FGFR1 punctate signal, whereas boxes represent ± SD. Statistical analyses were performed with Kruskal–Wallis H test (*p < 0.05; **p < 0.005 and ***p < 0.001). ABC?

Next, we determined the effect of concomitant treatment of U2OS-R1 cells with FGF1 and galectins that displayed the strongest impact on FGFR1 internalization (galectin-1, -7 and -8). As seen in Fig. 4A, the simultaneous supplementation of cells with FGF1 and galectin-1 resulted in a similar efficiency of FGFR1 endocytosis to the single treatments with FGF1 or galectin-1. The concurrent supplementation of cells with FGF1 and galectin-7 fully abolished the inhibitory effect of galectin-7 on FGFR1 endocytosis (Fig. 4B). In contrast, the presence of galectin-8 largely blocked the stimulatory effect of FGF1 on FGFR1 internalization (Fig. 4C).

These data indicate that distinct extracellular galectins, no matter whether they interact directly with N-glycans of FGFR1 or not, influence the efficiency of FGFR1 endocytosis. Galectins that exert the strongest impact on FGFR1 cellular uptake (galectin-1, -7 and -8) are the ones that directly bind FGFR1. Importantly, among tested galectins only galectin-1 stimulates FGFR1 endocytosis, whereas six other galectins (galectin-4, -7, -8, -9, -10, -13) to different extent inhibit FGFR1 internalization. Furthermore, our data imply that FGF1 can effectively overcome galectin-7-induced blockade of FGFR1 uptake, which is not the case for galectin-8.

### Multivalency of galectin-1 is not essential for the induction of CME of FGFR1

**Fig. 5:**
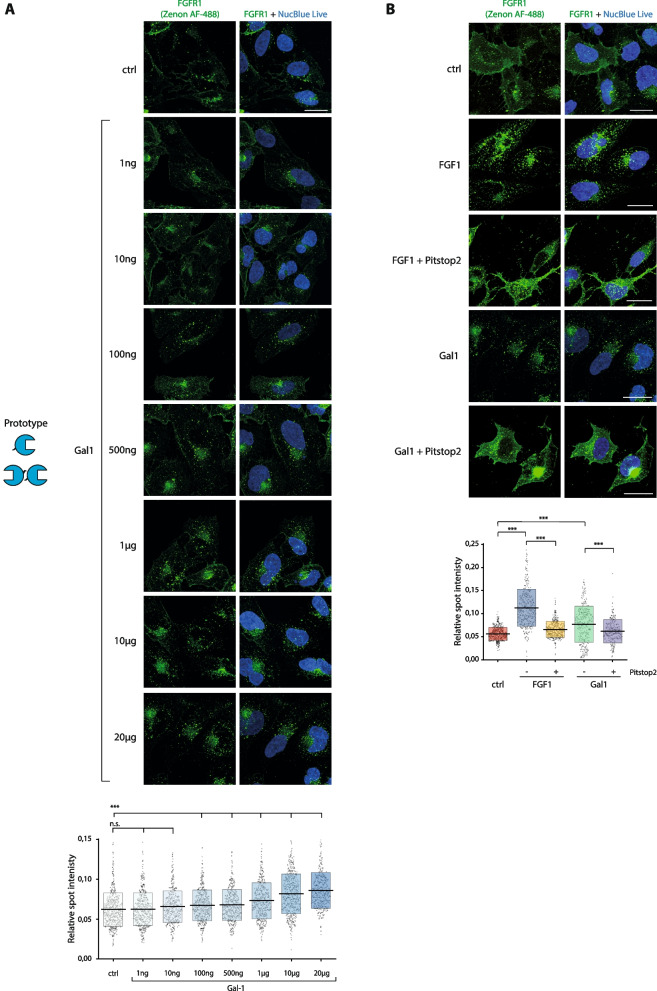
Galectin-1 induces CME of FGFR1. **A** Serum-starved, lactose-washed U2OS-R1 cells were treated with increasing concentrations of galectin-1 (1 ng/ml—20 μg/mL) for 30 min at 37 °C. **B** U2OS-R1 cells were preincubated with DMSO or Pitstop2 (30 μM) for 15 min at 37 °C. Next, cells were treated with galectin-1 (20 μg/mL) and intracellular FGFR1 was measured with the quantitative confocal microscopy. Representative images from at least three independent experiments are shown. Scale bars represent 20 μm. Each grey spot in the graph represents relative intracellular punctate signal intensity of FGFR1 in the single cell. At least 385 cells for each condition from three independent experiments were measured. Horizontal lines in the graph represent average intensity of intracellular FGFR1 punctate signal, whereas boxes represent ± SD. Statistical analyses were performed with Kruskal–Wallis H test (*p < 0.05; **p < 0.005 and ***p < 0.001)

Next, we focused on the stimulatory activity of galectin-1 on FGFR1 endocytosis. Initially, we determined whether the efficiency of FGFR1 internalization triggered by galectin-1 depends on the concentration of the extracellularly administered galectin-1. As shown in Fig. 5A, galectin-1 impact on FGFR1 internalization was concentration-dependent and already low concentrations of galectin-1 (below 100 ng/mL) caused a significant induction of FGFR1 endocytosis.

To shed some light on the mechanism responsible for the galectin-1-mediated enhanced cellular uptake of FGFR1, we pretreated U2OS-R1 cells with Pitstop2, a specific inhibitor of CME, and measured the efficiency of FGFR1 endocytosis upon supplementation of model cells with FGF1 or galectin-1. The quantitative confocal microscopy experiments revealed a significant reduction of FGF1-mediated FGFR1 endocytosis in the presence of Pitstop2, which is in agreement with role of FGF1 in CME of FGFR1 (Fig. 5B) [26, 38]. Similarly, the galectin-1-dependent stimulatory effect on FGFR1 cellular uptake was largely abolished by Pitstop2, causing accumulation of FGFR1 on the cell surface (Fig. 5B). These data indicate that endocytosis of FGFR1 triggered by the extracellular galectin-1 occurs via CME.

**Fig. 6:**
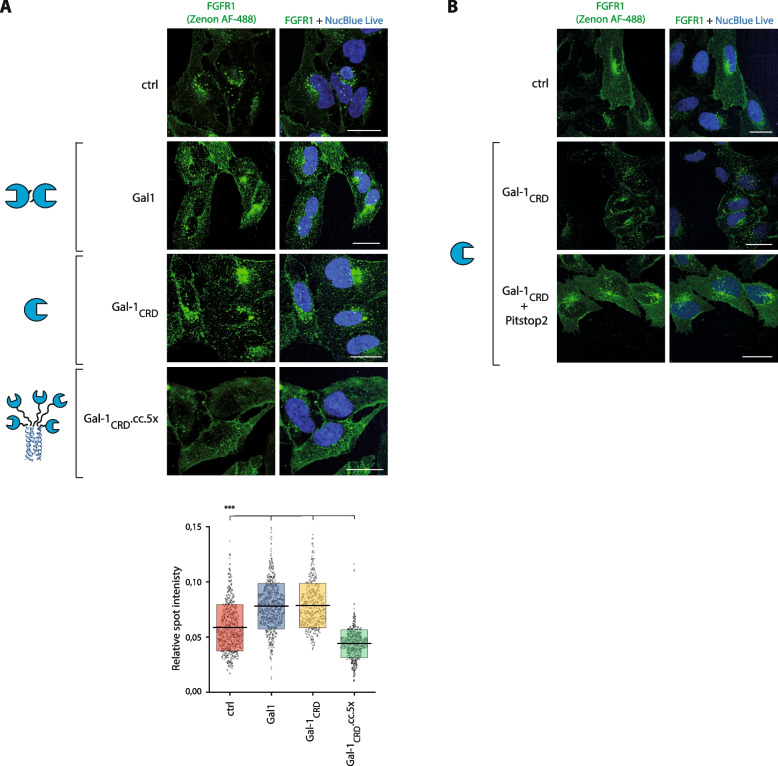
The valency determines the endocytic potential of galectin-1. **A** Serum-starved lactose-washed U2OS-R1 cells were incubated with the wild type galectin-1, galectin-1_CRD_ and gal-1_CRD_.CC.5x (20 μg/mL) for 30 min at 37 °C and the efficiency of FGFR1 endocytosis was assessed with the quantitative confocal microscopy. **B** U2OS-R1 cells were preincubated with DMSO or Pitstop2 (30 μM) for 15 min at 37 °C. Next, cells were treated with galectin-1_CRD_ (20 μg/mL) and efficiency of FGFR1 internalization was measured with the quantitative confocal microscopy. Representative images from at least three independent experiments are shown. Scale bars represent 20 μm. Each grey spot in the graph represents relative intracellular punctate signal intensity of FGFR1 in the single cell. At least 485 cells for each condition from three independent experiments were measured. Horizontal lines in the graph represent average intensity of intracellular FGFR1 punctate signal, whereas boxes represent ± SD. Statistical analyses were performed with Kruskal–Wallis H test (*p < 0.05; **p < 0.005 and ***p < 0.001)

Multivalency is critical for most of biological effects of galectins, especially for their extracellular and cell surface activities [49]. In accordance, we have recently demonstrated that the multivalency of galectins is essential for FGFR1 clustering, activation of the receptor and the initiation of FGFR1-dependent signaling pathways [47]. Galectin-1 is a prototype galectin composed of a single CRD that can dimerize with the use of an N-terminal motif, allowing for the multivalent interaction with ligands [58]. To determine the significance of the multivalency for galectin-1-mediated stimulation of FGFR1 CME, we made use of galectin-1_CRD_, a truncated variant of galectin-1 devoid of an N-terminal dimerization motif and thus incapable of multivalent interactions [47]. Importantly, Galectin-1_CRD_ is unable to trigger FGFR1 clustering and activation [47]. Galectin-1_CRD_ had virtually the same stimulatory effect on FGFR1 endocytosis as the wild type protein, indicating that dimerization of galectin-1 is not required for triggering CME of FGFR1 (Fig. 6A). Pretreatment of cells with Pitstop2 fully blocked the galectin-1_CRD_-dependent cellular uptake of FGFR1, indicating that the monovalent galectin-1_CRD_, similarly to the wild type multivalent galectin-1, stimulates CME of FGFR1 (Fig. 6B). Additionally, we made use of an engineered multivalent variant of galectin-1, gal-1_CRD_.CC.5x, in which oligomerization of galectin-1 CRD is facilitated by the pentamerizing coiled-coil sequence [47]. Interestingly, multivalent gal-1_CRD_.CC.5 × trapped FGFR1 on the cell surface, resulting in a significant inhibition of FGFR1 endocytosis (Fig. 6A).

These data indicate that galectin-1 stimulates CME of FGFR1 and that the multivalency of galectin-1 is not important for its effects on FGFR1 internalization. Our results also imply that by altering the valency of galectin-1 it is possible to fully reprogram its effect on FGFR1 endocytosis, changing galectin-1 from the efficient CME inducer to the potent endocytic blocker.

### Alterations in the molecular architecture of galectin-8 allow for fine-tuning FGFR1 endocytosis

**Fig. 7:**
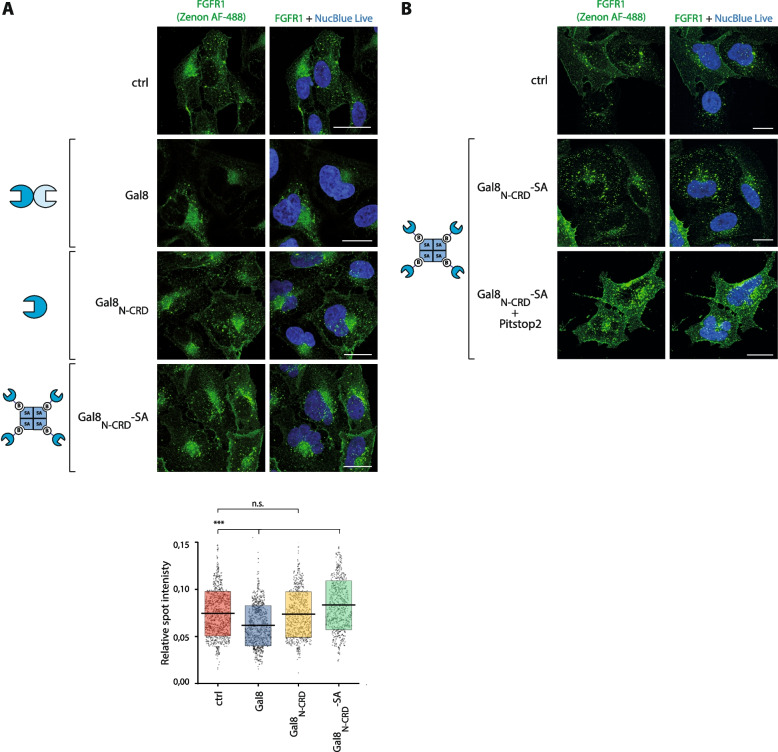
Engineering of galectin-8 from the endocytic inhibitor to the activator of FGFR1 cellular uptake. **A** Serum-starved lactose-washed U2OS-R1 cells were incubated with wild type galectin-8, recombinant monovalent Avi-Tagged variant of galectin-8: gal-8_N-CRD_ and its engineered tetravalent form assembled with the recombinant streptavidin (SA): gal-8_N-CRD_-SA (20 μg/mL) for 30 min at 37 °C. **B** Serum-starved U2OS-R1 cells were preincubated with DMSO or Pitstop2 (30 μM) for 15 min at 37 °C and cells were treated with gal-8_N-CRD_-SA (20 μg/mL). The efficiency of FGFR1 internalization was measured with the quantitative confocal microscopy. Representative images from at least three independent experiments are shown. Scale bars represent 20 μm. Each grey spot in the graph represents relative intracellular punctate signal intensity of FGFR1 in the single cell. At least 681 cells for each condition from three independent experiments were measured. Horizontal lines in the graph represent average intensity of intracellular FGFR1 punctate signal, whereas boxes represent ± SD. Statistical analyses were performed with Kruskal–Wallis H test (*p < 0.05; **p < 0.005 and ***p < 0.001)

Galectin-8 is a tandem repeat galectin composed of two distinct CRDs, from which only the N-terminal CRD directly binds FGFR1 [47]. To study if the multivalency is critical for galectin-8 inhibitory activity on the cellular uptake of FGFR1 we made use of recombinant monovalent Avi-Tagged variant of galectin-8: gal-8_N-CRD_ and its engineered tetravalent form assembled with recombinant streptavidin (SA): gal-8_N-CRD_-SA [47]. The quantitative confocal microscopy experiments revealed that in contrast to the wild type galectin-8, the monovalent engineered gal-8_N-CRD_ fully lost its ability to block FGFR1 endocytosis (Fig. 7A). The engineered SA-based tetravalent form of gal-8_N-_CRD, gal-8_N-CRD_-SA, significantly enhanced endocytosis of FGFR1 (Fig. 7A). Inhibition of CME with Pitstop2 fully abolished the stimulatory effect of the tetravalent gal-8_N-CRD_-SA on FGFR1 endocytosis (Fig. 7B).

These data demonstrate that multivalency of galectin-8 is crucial for galectin-8-mediated inhibition of FGFR1 endocytosis. Furthermore, our results indicate adjustments of galectin-8 valency allows for transformation of galectin-8 from the factor blocking FGFR1 internalization to the agent promoting CME of FGFR1.

### Galectins, by modulating FGFR1 endocytosis, differentially affect the kinetics of FGFR1 signaling

FGF binding to FGFR1 triggers activation of the receptor and initiation of downstream signaling cascades. Subsequent endocytosis of activated FGFR1 serves as a major cellular mechanism for the downregulation of FGFR1 signaling, as endocytosed FGF/FGFR1 is mostly directed towards lysosomes for degradation and the signal termination [18, 26, 31]. This mechanism prevents the prolonged signal transduction that may ultimately contribute to oncogenesis [19, 59]. We have recently shown that galectin-1, -3, -7 and -8 induce FGFR1 clustering on the cell surface, resulting in FGFR1 activation [47]. In the light of the prominent and opposite effects of galectin-1, -7 and -8 on FGFR1 endocytosis, we wondered if the altered cellular trafficking of FGFR1 will be reflected in modified kinetics of the signal transduction by the receptor.

**Fig. 8:**
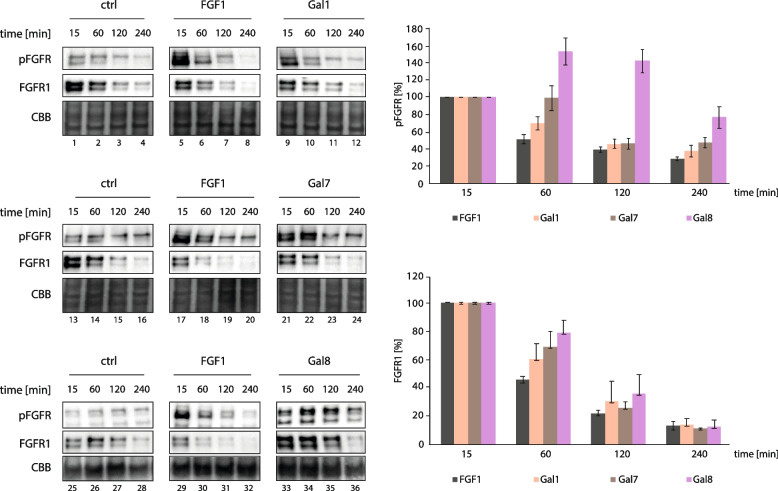
The kinetics of FGFR1 signaling and degradation upon stimulation of cells with galectins. Serum starved NIH3T3 cells were treated with cycloheximide to inhibit synthesis of new FGFR1 pool and incubated with galectins (20 μg/mL) or FGF1 (100 ng/mL) for various time points. Control shows untreated cells. Cells were lysed and the level of tyrosine-phosphorylated FGFR (pFGFR) and total FGFR1 in time upon incubation with FGF1 (positive control) or tested galectins was determined with western blotting. CBB served as a loading control (left panel). Densitometric analyses of the effect of galectins on pFGFR and FGFR1 signals (right panel). Data shown in the graphs are mean normalized pFGFR and FGFR values from at least three independent experiments ± SEM

To this end, we pretreated serum-starved cells with cycloheximide to block the synthesis of new FGFR1 molecules and determined the level of tyrosine-phosphorylated FGFR1 (pFGFR) and total FGFR1 in time upon incubation with FGF1 (positive control) or tested galectins. As shown in Fig. 8, supplementation of cells with FGF1 caused fast and efficient activation of FGFR1, seen as an immense increase in pFGFR signal at 15 min of stimulation. The signal of pFGFR rapidly decreased already after 1 h of stimulation with FGF1 and virtually reached the level of the non-stimulated control after 2 h (Fig. 8). Galectin-1 induced FGFR1 activation at 15 min of the treatment, but to lower extent than FGF1, which is in agreement with our previous findings (Fig. 8) [47]. The kinetics of pFGFR signaling upon incubation of cells with galectin-1 largely resembled the effects observed for FGF1, but with slightly slower disappearance of pFGFR signal in time (Fig. 8). Galectin-7 and -8, which both impede FGFR1 endocytosis, displayed largely altered kinetics of FGFR1 signaling in relation to FGF1. Galectin-7 exhibited prolonged activation of FGFR1, keeping pFGFR level at around 100% for up to 1 h, which was followed by a rapid decrease in pFGFR signal (Fig. 8). In contrast, galectin-8 induced phosphorylation of FGFR1 already after 15 min of stimulation, but reached the maximum of FGFR1 activation after 1 h (Fig. 8). Furthermore, the pFGFR signal persisted at the high level for much longer, with over 80% of maximal pFGFR level after 2 h and has not decreased to the level of the control in the studied period (Fig. 8). The altered kinetics of FGFR signaling upon treatment of cells with galectin-8 was also detected at the level of ERK-downstream target, p90RSK (Fig. S1). To assess the contribution of studied galectins to the receptor degradation (which occurs mostly via endocytosis and lysosomal degradation), we also monitored the total FGFR1 levels in time upon the treatment of cells with FGF1, galectin-,1, -7 and -8. As expected, supplementation of cells with FGF1 caused rapid degradation of FGFR1, indicating trafficking of CME-internalized FGF1/FGFR1 to lysosomes and their subsequent proteolysis (Fig. 8). Similar kinetics of FGFR1 degradation were observed for cells supplemented with galectin-1 (Fig. 8). In contrast, the stimulation of cells with galectin-7, and especially galectin-8, resulted in sustained levels of FGFR1 (Fig. 8).

These data indicate that the differential modulation of FGFR1 endocytosis by galectin-1, -7 and -8 is reflected in the kinetics of FGFR1 signaling and degradation. Both FGF1 and galectin-1 directly activate FGFR1 and after short and intensive pulse of FGFR1 signaling, the receptor is shut down due to the induction of CME by FGF1 or galectin-1, followed by lysosomal degradation of the receptor. Galectin-7 and -8 also directly activate FGFR1 by the receptor clustering mechanism, but by inhibiting FGFR1 endocytosis and degradation, these galectins largely prolong FGFR1 signaling.

## Discussion

Cell signaling and endocytosis are processes that are tightly and bidirectionally linked in human cells [60, 61]. Galectins emerged in the last years as major regulators of endocytosis and signaling [45, 49, 62]. On one hand, members of galectin family are powerful stimulators of endocytosis and master regulators of a specific glycolipid–lectin (GL–Lect) endocytic pathway, in which galectins and glycoconjugates facilitate formation of clathrin-independent carriers (CLICs) containing specific cargoes [22, 49, 63–66]. GL-Lect pathway was so far demonstrated only for galectin-3 and -8 and few specific cargoes. Galectin-8 interacts at the cell surface with a heavily glycosylated CD166 and utilizes GL-Lect mechanism to drive internalization of CD166 with the aid of Endophilin A3 [56, 67]. GL-Lect pathway is also employed by galectin-3 to trigger endocytosis of CD44 and β1-integrin [57, 68]. Besides GL-Lect, galectin-3 was implicated in macropinocytosis in glioblastoma stem cells [69]. Galectin family members can also modulate endocytosis of other cell surface proteins, but the underlying molecular mechanism is unknown. For example, galectin-3 mediates endocytosis of mucin type O-glycosylated Wnt signaling receptor frizzled-5 (Fzd5) in mouse embryonic stem cells. Galectin-3 stimulates the cellular uptake of lipopolysaccharide (LPS) and pandemic norovirus [70–72]. In neurons, galectin-1 promotes endocytosis of PlexinA4 receptor and this effect requires complex branched N-glycans of the receptor [73]. In megakaryocytes galectin-8 promotes endocytosis of coagulation factor V [74].

On the other hand, the multivalent binding of glycosylated cell surface protein by galectins may lead to the assembly of the galectin lattice that immobilizes proteins on the cell surface [75–77]. For example, in pancreatic beta cells galectin-2 traps cationic amino acid transporter in the galectin-2-enriched lattice containing also teneurin-3 [78]. In metastatic colon adenocarcinoma cells galectin-3 impedes endocytosis of death receptors, thus blocking cells response to tumor necrosis factor-related apoptosis-inducing ligand, TRAIL, and inhibiting apoptosis [79]. Galectin-3 stabilizes EGFR and TGFβR on the cell surface and by this modulates their signaling outputs [80]. At epithelial cell surface, galectin-7 stabilizes E-cadherin by blocking its endocytosis [81]. Galectin-8 was shown to negatively regulate internalization of insulin receptor (IR) [82]. Although it is evident that galectins can act both as activators or inhibitors of endocytosis, our knowledge on the role of particular galectin family members in endocytosis is still largely limited.

**Fig. 9:**
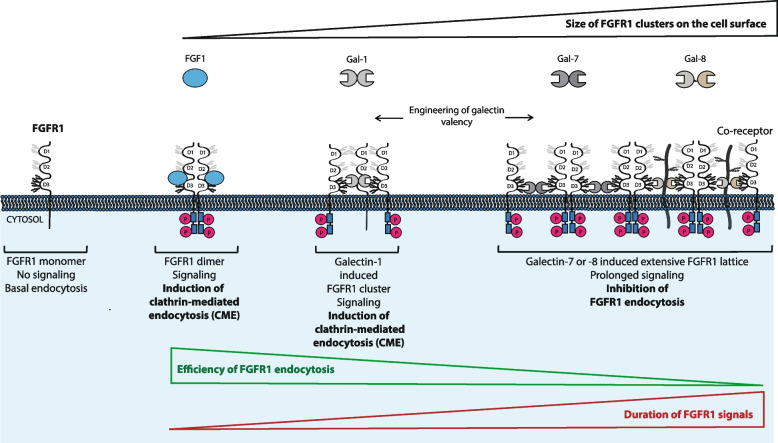
The hypothetical model of the significance of galectins multivalency for the modulation of FGFR1 endocytosis and signaling. Inactive monomeric FGFR1 is taken up by cells at low basal levels. FGF1 induces dimerization of FGFR1, activates FGFR1 and induces CME of the receptor that directs FGFR1 to lysosomes for degradation, downregulating FGFR1 signals. Galectin-1 mediates crosslinking of FGFR1 into smaller clusters, inducing clustering-based activation of FGFR1 followed by CME and lysosomal degradation of FGFR1, terminating the signals. Extensive clustering of FGFR1 on the cell surface by galectin-7 or galectin-8, which likely includes additional proteins like FGFR1 co-receptors, causes inhibition of FGFR1 endocytosis and concomitant clustering-based activation of FGFR1. Galectin-activated FGFR1 transduces signals for extended time, as lattice-trapped FGFR1 is deficient in endocytosis. Engineering of galectins valency allows for transformation of galectins from endocytic inhibitors to activators and vice versa

We have recently performed a comprehensive interaction study between the human galectin family members and FGFRs, and demonstrated that galectin-1, -3, -7 and -8 directly bind N-glycans of the D3 domain of the extracellular region of FGFR1, causing the clustering of FGFR1 and subsequent activation of the receptor [46, 47]. Since endocytosis of FGFR1 usually follows FGFR1 activation and constitutes a major mechanism for the receptor shutdown, we decided to study in depth the cellular trafficking of FGFR1 in the presence of galectins. We observed that among tested galectins, only galectin-1 was able to stimulate FGFR1 endocytosis. Using specific inhibitor od CME, we have shown that galectin-1 induced internalization of FGFR1 occurs via CME, similarly to the effect triggered by FGF1 (Fig. 9). Galectin-1 is a prototype galectin that is capable of multivalent binding of ligands supported by dimerization via the N-terminal unstructured tail [58, 83]. We have recently demonstrated that the monovalent variant of galectin-1, devoid of an N-terminal dimerization motif, and thus composed solely of CRD (galectin-1_CRD_), was unable to activate FGFR1 [47]. Furthermore, we were able to restore galectin-1_CRD_-mediated FGFR1 activation just by increasing its valency via the fusion of galectin-1_CRD_ with the pentamerizing coiled coil motif [47]. These data support the model in which FGFR1 clustering by galectin-1 constitutes the mechanism of FGFR1 activation (Fig. 9) [47]. On the other hand, monovalent galectin-1_CRD_ triggered CME of FGFR1 with virtually the same efficiency as the wild type galectin-1, whereas the pentavalent gal-1_CRD_.CC.5x (efficiently activating FGFR1) strongly inhibited FGFR1 internalization [47]. We hypothesize that the monovalent interaction between galectin-1_CRD_ and N-glycans of the D3 domain of FGFR1 triggers receptor oligomerization (or stabilizes preexisting FGFR1 oligomers), trapping the receptor in a kinase-inactive state that is competent to recruit endocytic adaptor proteins and initiate CME of FGFR1 (Fig. 9) [84]. The wild type galectin-1 likely does the same as galectin-1_CRD_, but due to the bivalency, additionally crosslinks FGFR1 into smaller clusters, allowing for simultaneous FGFR1 activation and subsequent uptake of the activated receptor by CME (Fig. 9). In line with these assumptions, an extensive FGFR1 crosslinking with the pentavalent gal-1_CRD_.CC.5 × results in an efficient clustering-based activation of the receptor, but in this situation the FGFR1 clusters are likely to extensive for endocytic machineries, thus impeding FGFR1 internalization (Fig. 9) [47]. Our data are in agreement with the stimulatory effect of galectin-1 on PlexinA4 receptor endocytosis in neurons, yet in that case the mechanism of galectin-1-triggered endocytosis and the significance of galectin-1 multivalency are unknown [73]. Collectively, these data implicate that the impact of galectin-1 on FGFR1 endocytosis is uncoupled from the receptor signaling and that it is possible to alter the endocytic potential of galectin-1 by changing its valency (Fig. 9). We have observed similar phenomenon with anti-FGFR1 antibody fragments that triggered FGFR1 dimerization and CME without activation of the receptor [26].

We have recently demonstrated that galectin-3, -7 and -8 cause an efficient clustering-based activation of FGFR1 and that this effect is fully dependent on galectins multivalency [47]. Here, we show that besides activating FGFR1, galectin-7 and -8 inhibit endocytosis of the receptor. This in turn results in the stabilization of FGFR1 on the cell surface and prolonged signaling of galectin/FGFR1 in relation FGF1/FGFR1 (Fig. 9). Galectin-8 is a tandem-repeat galectin that contains two distinct CRDs on a single polypeptide chain and only the N-terminal CRD of galectin-8 directly binds FGFR1 [47, 85]. We assume that the wild type galectin-8 uses the N-terminal CRD to bind N-glycans of FGFR1, whereas the C-terminal CRD binds other yet unknown cell surface protein/s (likely FGFR1 binding partner). This traps FGFR1 on the cell surface in the heterogenous lattice, causing the clustering-based FGFR1 activation and at the same time impedes FGFR1 endocytosis (Fig. 9). Furthermore, the engineering of galectin-8 towards the monospecific tetravalent variant, gal-8_N-CRD_-SA, transformed galectin-8 from the effective endocytic inhibitor into the stimulator of FGFR1 internalization via CME. We hypothesize that this metamorphosis is due to the smaller clusters of FGFR1 triggered by the gal-8_N-CRD_-SA variant and their homogenous composition (Fig. 9). In line with these observations, using multivalent FGFR1 ligands of different architecture we have demonstrated that whereas controlled clustering of FGFR1 results in the highly efficient FGFR1 internalization via multiple endocytic pathways, too extensive FGFR1 crosslinking inhibits uptake of the receptor [40–43, 86]. Formerly, we have observed inhibition of FGFR1 internalization by the extracellular galectin-3 [46]. Here, we confirmed these findings, but the effect was minor and thus below statistical thresholds which was likely caused by the different experimental setup, as in this study we depleted cells of endogenous galectins and probed the effect of particular galectin family members, which was not the case in the previous study. These data indicate that the composition of galectin lattice may influence the final effect of galectins on endocytosis. This issue requires clarification in the future.

The mechanisms by which galectins themselves can enter cells are still mysterious. It was reported that depending on type of macrophages galectin-3 is endocytosed either in carbohydrate dependent or independent manner [87]. In T-cells galectin-1 is internalized together with CD7 receptor and GM1 ganglioside via clathrin-mediated and clathrin-independent endocytosis [88]. Here we demonstrate that all extracellularly administered galectins (no matter whether they directly bind FGFR1 or not) partially co-localize with FGFR1, implicating that to some extent galectins, directly or not, employ FGFR1 for the cell entry. Clearly further studies are needed for the identification of endocytic receptors for particular galectins and for elucidating their intracellular fate after endocytosis. The restriction of our study is that in microscopy experiments we have used only one cellular model, U2OS, with FGFR1 overproduction, which may influence the glycosylation status of the receptor and by this partially limit generality of the conclusions presented.

Summarizing, our data provide novel insights into the interplay between cellular trafficking and signaling. We show that specific galectins directly activate FGFR1 by clustering-based mechanism and the size of FGFR1 clusters seems to determine the efficiency of FGFR1 endocytosis. Our data implicate that galectin-mediated cross-linking of FGFR1 into small clusters (up to four receptor molecules) activates FGFR1 and induces receptor endocytosis by CME, leading to rapid signal termination, whereas the assembly of extensive FGFR1 clusters (over five FGFR1 molecules) supported by galectins multivalency traps FGFR1 on the cell surface in the active state and prolongs cellular signaling (Fig. 9). Since galectins and FGFR1 are strongly implicated in cancers, our data might contribute to the development of novel anti-cancer therapeutic strategies [10, 52]. Furthermore, we provide evidence that it is possible to fine-tune galectin-1 and -8 signaling and endocytic activities by altering their valency (Fig. 9). Similar observations were recently made for galectin-3 [89]. Due to the large potential of galectins in biotechnology, our data might allow for engineering of galectins with preferable cellular activities [90].

## Materials and methods

### Antibodies and reagents

The primary antibodies directed against FGFR1 (#9740), p90RSK (Ser380, #11,989) and phospho-FGFR (pFGFR; #3476), were from Cell Signaling (Danvers, MA, USA). Secondary antibodies were obtained from Jackson Immuno-Research Laboratories (Cambridge, UK). The primary antibodies anti-EEA1 was from Cell Signaling (Danvers, MA, USA). Goat anti-rabbit AF-594 secondary antibody used for EEA1 detection was from Thermo Fisher Scientific (Waltham, MS, USA). NucBlue Live, DyLight™ 550 NHS Ester, Zenon AF-488, CellMask™ Plasma Membrane stains were from Thermo Fisher Scientific (Waltham, MS, USA). Pitstop2 was from Sigma-Aldrich (St. Louis, MO, USA).

### Cell culture

U2OS cells stably expressing FGFR1 (U2OS-R1) were obtained by transfection of U2OS cells with expression plasmid encoding FGFR1 as described in [10]. Cells were cultured in 5% CO_2_ atmosphere at 37 °C in Dulbecco’s Modified Eagle’s Medium (Biowest, Nuaille, France) supplemented with 10% fetal bovine serum (FBS) (Thermo Fisher Scientific), antibiotics mix (100 U/mL penicillin and 100 μg/mL streptomycin) (Thermo Fisher Scientific), for U2OS-R1 additionally supplemented with 1 mg/mL geneticin (Thermo Fisher Scientific). Mouse embryo fibroblast cells (NIH3T3) were cultured in Dulbecco’s Modified Eagle’s Medium—DMEM (Thermo Fisher Scientific) supplemented with 10% fetal bovine serum (FBS) (Thermo Fisher Scientific) and antibiotics (100 U/mL penicillin, 100 μg/mL streptomycin). Cells were seeded onto tissue culture plates one day before the experiments.

### Recombinant proteins

Human recombinant galectins were expressed and purified as described previously [47]. Recombinant human galectins were fluorescently labeled with DyLight™550 NHS dye according to manufacturers’ protocol (#62,263, Thermo Fisher Scientific, United States). T-Fc which is a recombinant antibody recognizing FGFR1, was produced as described in [40].

### Kinetics of FGFR1 signaling

To determine the kinetics of FGFR1 signaling and degradation, NIH3T3 cells were starved overnight (12-well plates, 100 000 cells/well) and then treated with cycloheximide (10 μg/mL), FGF1 (100 ng/mL) in the presence of heparin (10 U/mL) or galectin-1, -7 and -8 (20 μg/mL) at 37 °C. At distinct time points (15 min, 1 h, 2 h and 4 h) cells were lysed in Laemmli buffer and analyzed by SDS-PAGE and western blotting.

### Fluorescence microscopy

For analysis of galectin-DyLight550 co-localization with FGFR1, the serum starved U2OS-R1 cells were treated with 50 mM lactose for 15 min at 37 °C and fluorescently labeled galectins (20 μg/mL) were added to the cells, and incubated for 30 min at 37 °C. Next, cells were fixed in 4% paraformaldehyde and permeabilized with 0.1% Triton in PBS for 10 min, followed by the treatment with anti-FGFR1 antibody T-Fc (15 μg/mL) for 30 min at 37 °C. Zenon-AF488 was used for visualization of the T-Fc and NucBlue Live was used for fluorescent labeling of nuclei. For co-localization of FGFR1 with an early endosome marker protein, serum starved lactose-treated U2OS-R1 cells were treated with galectins (20 μg/mL) and FGF1 (100 ng/mL) for 30 min at 37 °C. Cells were fixed in 4% paraformaldehyde, permeabilized in 0.1% Triton X-100 and then treated with T-Fc (15 μg/mL) for 30 min at 37 °C. Zenon-AF488 was used for labeling of the T-Fc, NucBlue Live was used for fluorescent labeling od nuclei and cells were fixed again in 4% paraformaldehyde. Next, cells were blocked with 2% BSA for 30 min and incubated with rabbit anti-human polyclonal early endosome antigen 1 (EEA1) antibody (#ab2900, Abcam) and anti-rabbit IgG secondary antibody conjugated to Alexa Fluor 594 (#A11037, Thermo Fisher Scientific). The cells were analyzed with fluorescence microscopy. Wide-field fluorescence microscopy was carried out using a Zeiss Axio Observer Z1 fluorescence microscope (Zeiss, Oberkochen, Germany) as described in [91]. Images were processed with Zeiss ZEN 2.3 software (Zeiss, Oberkochen, Germany), and Adobe Photoshop CS6 (Adobe, San Jose, CA, USA).

For analyses of the efficiency of FGFR1 internalization, serum starved lactose-treated U2OS-R1 cells were incubated with recombinant galectins (20 μg/mL), FGF1 (100 ng/mL) or mixtures of studied proteins for 30 min at 37 °C, which was followed by FGFR1 staining as described above. To identify the mechanism responsible for the uptake of FGFR1, the serum starved U2OS-R1 cells were pretreated Pitstop2 (30 μM) or DMSO (control) for 15 min at 37 °C.

Fixed and labelled cells were analyzed with the quantitative confocal microscopy using the Opera Phenix Plus High-Content Screening System (Perkin Elmer, Waltham, MA, USA). Measurements were carried out using confocal mode with 63 × Water, NA 1.15 objective with binning 2 using two peaks autofocus. 37 fields per well were imaged, with 7 Z-stack per field at 0,5 μm interval to ensure comprehensive imaging of the cell. 2160 × 2160 px Camera ROI was used to capture the images. The Harmony High-Content Imaging and Analysis Software (version 5.1; Perkin Elmer, Waltham, MA, USA) was used for image acquisition and analysis. Number of cells and the cell boundaries were determined using the DAPI and the Cell Mask Deep Red, respectively. The intracellular punctate Zenon-AF488 signal was measured. Images were assembled in Illustrator (Adobe) with only linear adjustments of the contrast and brightness.

### Statistics

All of the experiments presented in the manuscript were repeated at least three times. Statistical analyses were performed with Kruskal–Wallis H test (*p < 0.05; **p < 0.005 and ***p < 0.001).

### Supplementary Information

Supplementary Material 1.

## Data Availability

Data are available from the corresponding author upon reasonable request.

## Abbreviations

BLI: Biolayer interferometry
CRD: Carbohydrate recognition domain
CBB: Coomassie brilliant blue
CC: Coiled coil
CLICs: Clathrin-independent carriers
CIE: Clathrin-independent endocytosis
CME: Clathrin medited endocytosis
DMEM: Dulbecco’s Modified Eagle’s Medium
EEA1: Early endosome antibody 1
FBS: Fetal bovine serum
FGFs: Fibroblast growth factors
FGFRs: Fibroblast growth factor receptors
IR: Insulin receptor
JM: Juxtamembrane domain
SA: Streptavidin
TK: Tyrosine kinase
