# Supplementary material for: The diverse dependence of galectin-1 and -8 on multivalency for the modulation of FGFR1 endocytosis
Source: Cell Commun Signal. 2024 May 15;22:270. doi: 10.1186/s12964-024-01661-3 (PMC11094976; doi:10.1186/s12964-024-01661-3)
Supplement: Supplementary file 1 — Supplementary Material 1. [file 12964_2024_1661_MOESM1_ESM.pdf]

Supplementary Information for

The diverse dependence of galectin-1 and -8 on multivalency for the modulation of FGFR1 endocytosis

Dominika Żukowska[1], Aleksandra Chorążewska[1], Krzysztof Ciura[1], Aleksandra Gędaj[1], Marta Kalka[1], Marta Poźniak[1], Natalia Porębska[1], and Łukasz Opaliński[1]*

[1]Department of Protein Engineering, Faculty of Biotechnology, University of Wroclaw, Joliot-Curie 14a, 50-383 Wroclaw, Poland

*Correspondence should be addressed to Ł.O (lukasz.opalinski@uwr.edu.pl)

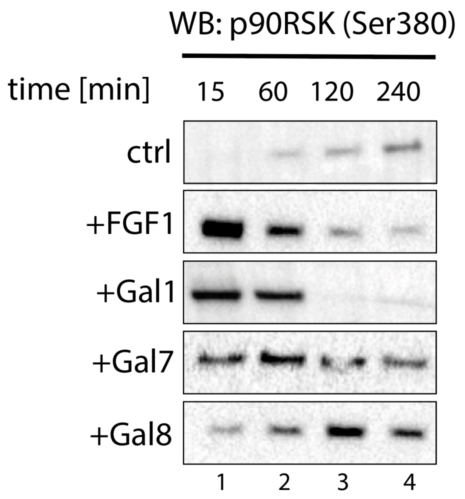

Fig. S1. The kinetics of p90RSK phosphorylation at serine 380 upon treatment of cells with FGF1 and selected galectins. Serum starved NIH3T3 cells were treated with cycloheximide to inhibit synthesis of new FGFR1 pool and incubated with galectins (20 µg/mL) or FGF1 (100 ng/mL) for various time points. Control shows untreated cells. Cells were lysed and the level of serine 380 phosphorylation of p90RSK was determined with western blotting.

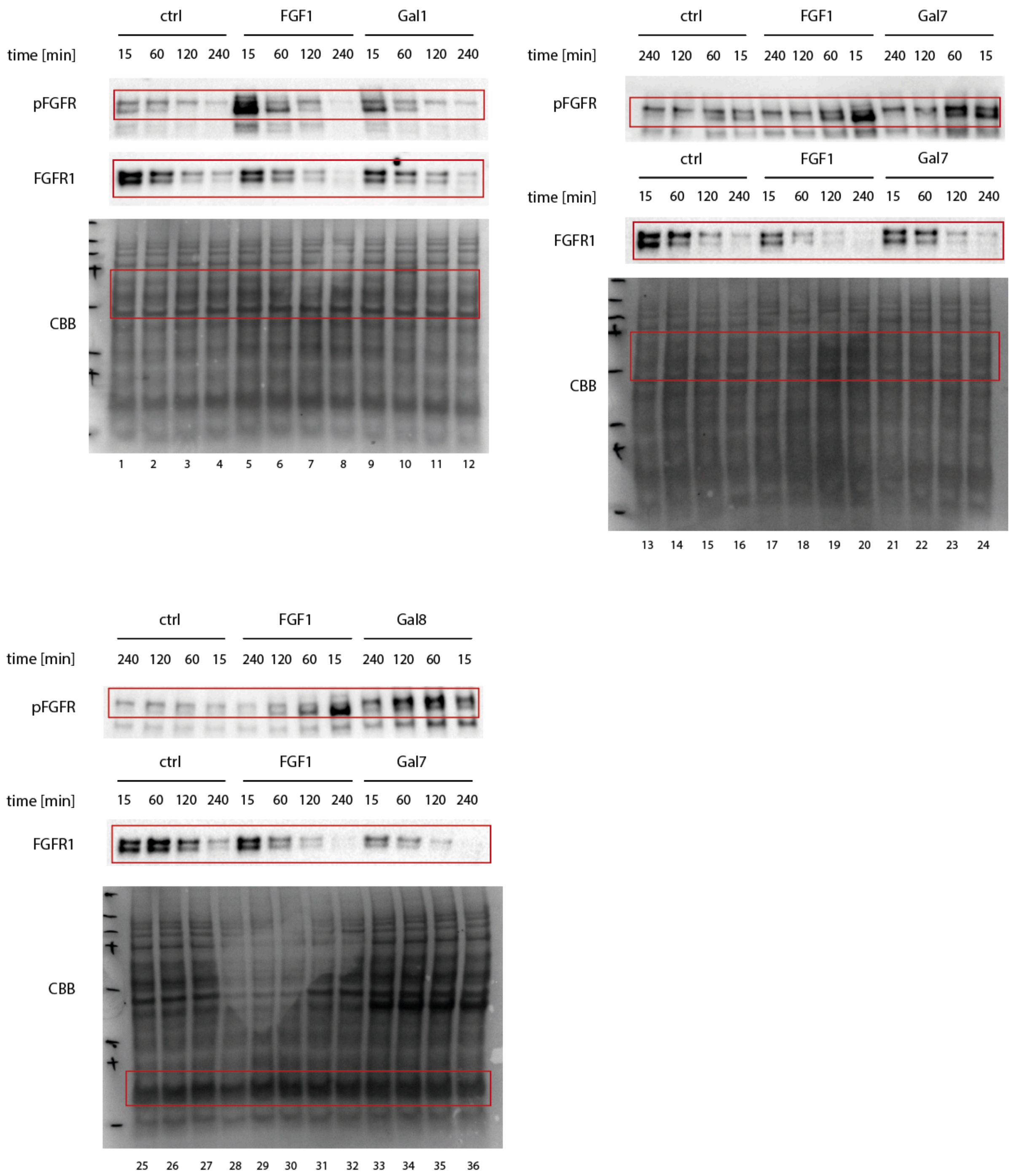

Fig. S2. Full length original blots used for preparation of the main figures.
